# Viability of the virtual cone technique using a fixed small multi‐leaf collimator field for stereotactic radiosurgery of trigeminal neuralgia

**DOI:** 10.1002/acm2.14148

**Published:** 2023-09-18

**Authors:** Taindra Neupane, Charles Shang, Maxwell Kassel, Wazir Muhammad, Theodora Leventouri, Timothy R. Williams

**Affiliations:** ^1^ Department of Physics Florida Atlantic University Boca Raton Florida USA; ^2^ RSO South Florida Proton Therapy Institute Delray Beach Florida USA; ^3^ Center for Biological and Materials Physics (CBAMP) Department of Physics Florida Atlantic University Boca Raton Florida USA; ^4^ Medical Director South Florida Proton Therapy Institute Delray Beach Florida USA

**Keywords:** dosimetric uncertainties, fixed virtual cone, Monte Carlo simulations, stereotactic radiosurgery, trigeminal neuralgia

## Abstract

Dosimetric uncertainties in very small (≤1.5 × 1.5 cm^2^) photon fields are remarkably higher, which undermines the validity of the virtual cone (VC) technique with a diminutive and variable MLC fields. We evaluate the accuracy and reproducibility of the VC method with a very small, fixed MLC field setting, called a fixed virtual cone (fVC), for small target radiosurgery such as trigeminal neuralgia (TGN). The fVC is characterized by 0.5 cm x 0.5 cm high‐definition (HD) MLC field of 10MV FFF beam defined at 100 cm SAD, while backup jaws are positioned at 1.5 cm x 1.5 cm. A spherical dose distribution equivalent to 5 mm (diameter) physical cone was generated using 10–14 non‐coplanar, partial arcs. Dosimetric accuracy was validated using SRS diode (PTW 60018), SRS MapCHECK (SNC) measurements. As a quality assurance measure, 10 treatment plans (SRS) for TGN, consisting of various arc ranges at different collimator angles were analyzed using 6 MV FFF and 10 MV FFF beams, including a field‐by‐field study (*n* = 130 fields). Dose outputs were compared between the Eclipse TPS and measurements (SRS MapCHECK). Moreover, dosimetric changes in the field defining fVC, prompted by a minute (± 0.5–1.0 mm) leaf shift, was examined among TPS, diode measurements, and Monte Carlo (MC) simulations. The beam model for fVC was validated (≤3% difference) using SRS MapCHECK based absolute dose measurements. The equivalent diameters of the 50% isodose distribution were found comparable to that of a 5 mm cone. Additionally, the comparison of field output factors, dose per MU between the TPS and SRS diode measurements using the fVC field, including ± 1 mm leaf shift, yielded average discrepancies within 5.5% and 3.5% for 6 MV FFF and 10 MV FFF beams, respectively. Overall, the fVC method is a credible alternative to the physical cone (5 mm) that can be applied in routine radiosurgical treatment of TGN.

## 1 INTRODUCTION

Dosimetry in small (≤ 3 cm x 3 cm) photon field is complex due to the presence of small‐field related conditions^1^: (1) Lack of lateral charged particle equilibrium (LCPE), (2) Partial occlusion of the primary radiation source by collimating devices, and (3) The field size is comparable or smaller than the detector size. The International Codes of Practice (CoPs) such as the IAEA TRS‐483^2^, ^3^ and AAPM TG‐155^4^ that provide the comprehensive guidelines for accurate and consistent small‐field dosimetry, are now available for reference and relative dose determination. In modern radiation therapy, the use of the small (≤ 3 cm x 3 cm)^1^ and very small (≤ 1.5 cm x 1.5 cm),^5^ non‐standard fields (static or dynamic) are very common for advanced treatments such as Intensity Modulated Radiation Therapy (IMRT),^6^ Volumetric Modulated Arc Therapy (VMAT),^7^ Stereotactic Radiosurgery (SRS),^8^ and Stereotactic Body Radiotherapy (SBRT)^9^ to treat relatively small lesions.

Currently, the cone‐based SRS using the specialized machines such as GammaKnife, CyberKnife, and other LINACs remains the standard practice for the treatment of small intracranial lesions such as trigeminal neuralgia (TGN).^10^, ^11^, ^12^, ^13^ However, it poses several challenges; need of (1) a cone‐specific dose calculation, (2) ensuring geometric precision for each cone collimating system, (3) rigorous patient‐specific quality assurance (QA), and (4) avoidance of collision for cone‐based arcs. It should be noted that a well‐defined spherical or ellipsoidal dose distribution along with a high mechanical precision in dose delivery is required for such treatment. It is also crucial to have the advanced LINAC that can deliver radiation beams with a sub‐millimeter accuracy in addition to the correctly defined outputs and accurate modeling of very small field/beam during the treatment planning. With the introduction of advanced MLC (leaf width 2.5−3.0 mm) technology, there is ongoing research on the MLC‐based SRS for the treatment small targets to achieve similar dosimetric goals in more efficient manner. To our best knowledge, the first MLC‐based radiosurgery for trigeminal neuralgia was reported and validated on the Varian TrueBeam STx LINAC^11^, ^14^ in which the 6 MV FFF beam was delivered with 18−21 static conformal fields (field size range, 0.6−0.7 cm). Next, a virtual cone technique using different standardized MLC fields, was introduced to replicate the dose distributions generated by physical cones. In this technique, very small non‐square fields defined by two central leaves of 10 MV FFF photon beams were used in concert with 7−10 non‐coplanar arcs to produce the spherical dose distribution comparable to 4−5 mm (diameter) physical cones.^15^, ^16^ However, the use of such diminutive and non‐square MLC fields could add further dosimetric uncertainties and complexities to the treatment.

It is evident that the effect of gravity on the MLC or imprecision of MLC leaves travel can cause notable dosimetric variation in small‐field (SRS) arc therapy in addition to gantry, collimator, couch rotations, and individual characteristics of leaves (shape, design).^9^, ^17^, ^18^ An infinitesimal leaf shift of ± (0.5−1.0 mm) is normally expected (depending on how good the QA is performed) during the gantry rotation and it is noteworthy when the gantry is at 90° or 270° angle and the collimator is at 0° angle.^19^, ^20^ As a result, meaningful dosimetric uncertainties can be observed due to the significant geometrical variation of MLC fields during the small‐field arc treatments.^21^, ^22^, ^23^ To mitigate these uncertainties, we propose the virtual cone technique with a fixed field geometry (i.e., 0.5 cm x 0.5 cm symmetric HDMLC field defined by two pairs of central leaves while backup jaws are positioned at 1.5 cm x 1.5 cm, defined at 100 cm SAD) in conjunction with multiple non‐coplanar arcs that is suitable for the small target radiosurgery of TGN. This approach intends to not only reduce undesirable geometrical (i.e., MLC) uncertainties as compared to non‐fixed field techniques but also make the treatment more convenient, reliable, and robust than previous approaches. Moreover, we perform the Monte Carlo (MC) study for small‐field virtual cone that can provide further authentication and evaluate dosimetric uncertainties associated with potential geometrical variation of MLC fields during the treatment (gantry, collimator rotation). An integration of proper uncertainty correction during the treatment planning could notably improve the accuracy and robustness small target radiosurgery.^22^, ^24^

The purpose of the research is to (1) study the viability of the fixed virtual cone (fVC) approach for the radiosurgical treatments of small targets such as trigeminal neuralgia, and (2) evaluate its dosimetric robustness considering potential MLC leaf shift (± 0.5−1.0 mm) during the treatment from measurements and MC calculations.

## 2 MATERIALS AND METHODS

### 2.1 Experimental

Dosimetric measurements were performed on the Varian Edge radiosurgery system (Varian Medical Systems, Palo Alto, California, USA) at South Florida Proton Therapy Institute (Delray Beach, Florida, USA). The LINAC is equipped with a HDMLC that is capable of delivering 6 MV FFF and 10 MV FFF photon beams with a sub‐millimeter accuracy.^25^ It was defined at 100 cm source to surface distance (SSD) with 1 cGy/MU at depth of maximum dose and calibrated at 95 cm SSD, 5 cm depth (100 cm SAD).^26^ The dose outputs measurement geometry consisted of an isocentric setting of 100 cm SAD with: (1) 95 cm SSD, 5 cm depth, and (2) 90 cm SSD, 10 cm depth in the water phantom.^3^, ^26^ The dose calculation was performed in the Eclipse treatment planning system (TPS) using the Acuros XB algorithm (version 16.1) with a grid size of 1 mm x 1 mm x 1 mm.^26^, ^27^

A separate machine model (fVC) for TGN treatments was created in the Eclipse TPS and commissioned as described by the AAPM TG‐101,^9^ TG‐119,^28^ and Varian Medical Systems guidelines.^26^ Previously measured dosimetric leaf gap (DLG) values were modified to improve the agreement between measurements and calculations for such a very small‐field IMRT treatments. The fVC model was not designated as an equivalent model in the Eclipse; thus, a machine override was required at the treatment console prior to beam delivery. The beam modeling (for both energy beams) was performed on the Eclipse TPS with an Acuros XB algorithm and validated by the corresponding absolute dose measurements using the SRS MapCHECK (Sun Nuclear Corporation, FL) The SRS MapCHECK consists of 1013 n‐type solid‐state diodes (SunPoint 2 diode with 0.48 × 0.48 mm^2^ cross‐section area, 0.007 mm^3^ active volume) arranged on a 77 mm x 77 mm array with inter‐detector spacing of 2.47 mm.^29^ It is designed to be compatible with an end‐to‐end phantom, StereoPHAN (Sun Nuclear Corporation, Florida, USA), which provides a freedom of interchanging the SRS MapCHECK and a film holder inside of it enabling comparison between both measurements without introducing positional uncertainty. The mechanical as well as dosimetric accuracy (i.e., end‐to‐end test) of the final treatment plan were also verified with SRS MapCHECK measurement using StereoPHAN. The SRS MapCHECK was considered as a standalone QA device for all measurements. A unique CT and MR protocol was applied during image acquisitions to meet the requirement of high precision dose delivery, thereby optimizing the image quality of tumor site.

#### 2.1.1 Fixed virtual cone technique

**FIGURE 1:**
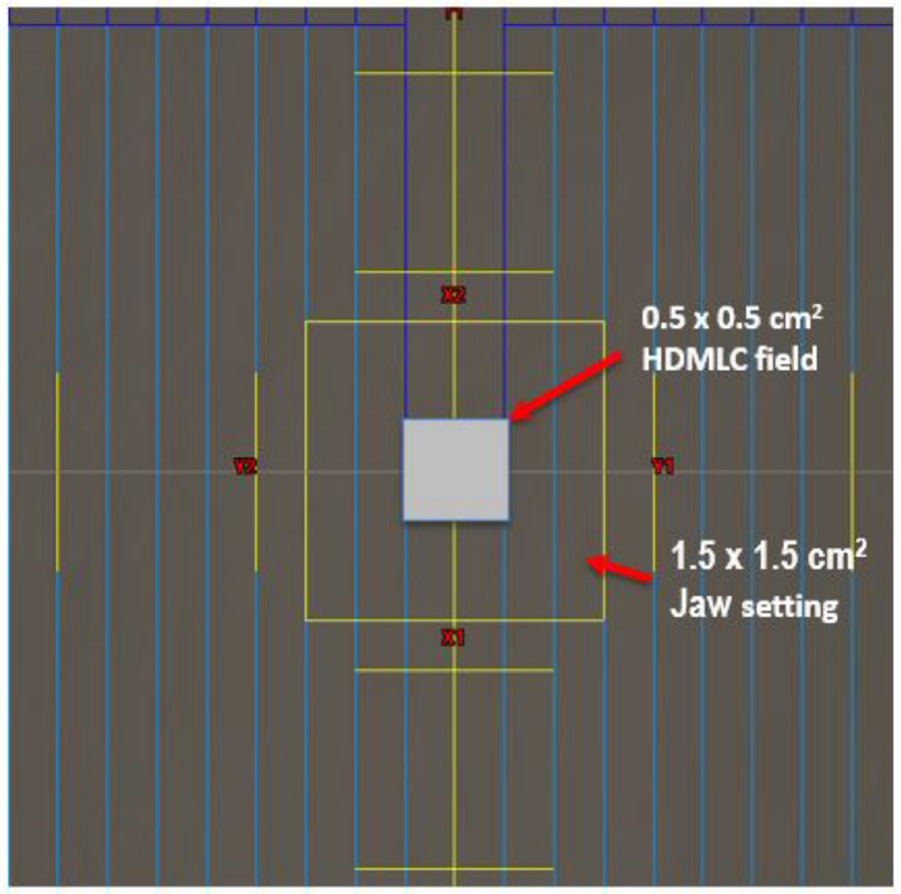
A beam eye view of the collimator settings for the fixed virtual cone (fVC) technique characterized by 0.5 cm x 0.5 cm HDMLC field, defined by two pairs of central leaves and fixed openings of jaws (1.5 cm x 1.5 cm) at 100 cm SAD.

**FIGURE 2:**
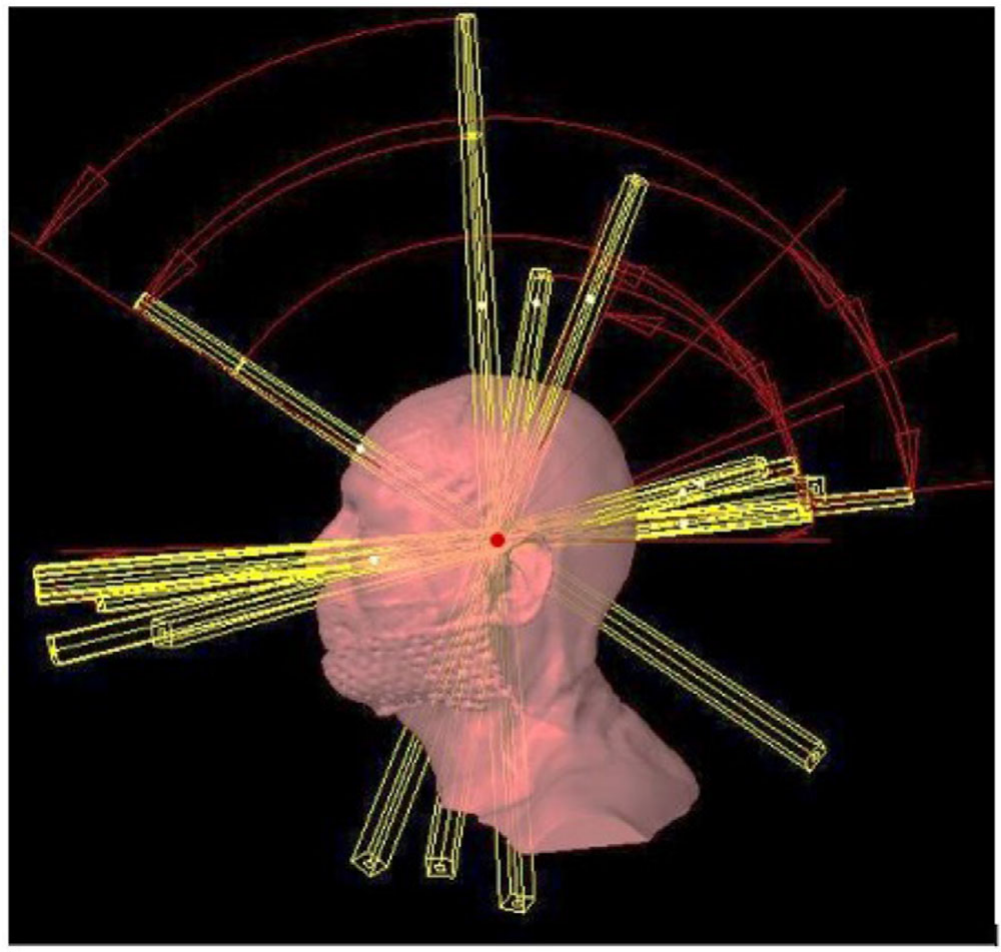
Three‐dimensional (3D) view of a treatment plan with multiple arc fields for the treatment of a very small tumor in left trigeminal nerve using the fixed (0.5 cm x 0.5 cm HDMLC field) virtual cone (fVC) technique.

We employed a virtual cone technique with a fixed field geometry, called a “fixed virtual cone (fVC) technique” to generate a very small, spherical dose distribution equivalent to 5 mm physical cone. The fVC was characterized by 0.5 cm x 0.5 cm HDMLC field at 100 cm SAD, defined by 2 pairs of central leaves while backup jaws were positioned at 1.5 cm x 1.5 cm (Figure 1). The spherical dose distribution was generated using several (10−14) partial arcs with different collimator angles and couch positions (Figure 2). For instance, at each couch position, different partial arcs with a clockwise and counter‐clockwise gantry rotation (gantry angle, 0°−360°, width, 30°−90°) were used with different collimator angles (0°−90°). The remaining leaves were positioned at 3 cm outside of the secondary collimation to avoid any geometrical uncertainties (photons attenuation). The 10 MV FFF photon beam was chosen clinically because of its high dose rate (up to 2400 MU/min) allowing for steep dose gradients, low collimator scatter, energy spectrum, and ability for SRS treatments to be delivered with a single fraction. When performing a high precision SRS for trigeminal neuralgia, the dose was delivered using multiple partial arcs at different collimator angles to optimize the dose coverage, thereby minimizing the dose to critical organs (brainstem). The dose per degree of the arcs was non‐uniform and proportional to the sine of the gantry angle. The weighting of the arcs was chosen in such a way that in the limit of large N and 2⊓ irradiation, it results in uniform fluence per unit area that is ideal for generating a spherical dose distribution.

#### 2.1.2 Patient‐specific quality assurance

The treatment plans (10 MV FFF) of 10 clinical cases (left, right trigeminal neuralgia with a prescribed dose 7000−8000 cGy per fraction) were selected for the verification process or quality assurance and re‐planned with the 6 MV FFF (dose rate 1400 MU/min) photon beams. The verification process consisted of the gamma evaluation of planar dose profiles through isocenter using global criteria of 3%/1 mm (field‐by‐field), 2%/1 mm (cumulative dose), and the comparison of dose outputs or dose/MU between the TPS and the SRS MapCHECK (Sun Nuclear Corporation, Florida, USA) measurements at 100 cm SAD using a spatial resolution of 0.10 cm for both energy beams. The dose parameters were compared on the field‐by‐field basis as well as whole plan using 10% threshold. The field‐by‐field study included the comparison of dose outputs (at central axis) for 130 fixed virtual cone fields with different arcs (width 30°−90°), collimator angles (0°−90°), and couch positions. It is noted that SSD was changing throughout the measurements (arc therapy) due to the elliptical cranial shape thereby keeping the same SAD of 100 cm. The collimator was rotated for differing fields prior to irradiation of the arcs to deliver uniform dose and reduce MLC transmission. Due to the leaf ends being rounded shape, the rounded leaf edges were moved outside the jaw settings to Bank A, where the jaw collimators shielded radiation for the MLC.

#### 2.1.3 TGN cases study

**FIGURE 3:**
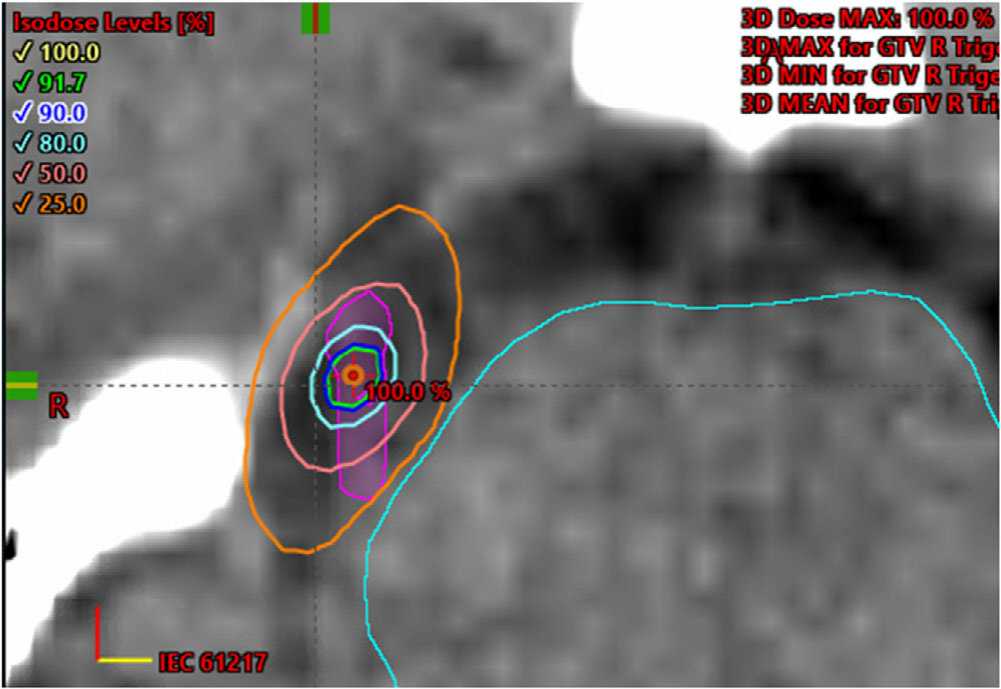
A representation of a treatment plan for trigeminal neuralgia using the fixed virtual cone technique along with multiple isodose lines. The isodose lines correspond to 90% (blue color), 50% (red‐orange color), and 25% (orange color) of the maximum dose are also displayed. A critical organ (i.e., brainstem) is contoured with a light blue color (left to the target).

Fifteen clinical cases treated for TGN (prescribed dose, 7000−8000 cGy) using the fVC method (10 MV FFF beam) were collected retrospectively and important clinical parameters such as number of arcs, mean and maximum doses to the target, and maximum dose at critical organ (brainstem) were recorded from treatment plans. Similarly, the irradiated area/volume encompassed by 90%, 50%, and 25% isodose curves (Figure 3) together with gamma indices (passing rate, criteria) were manually acquired from the SRS MapCHECK QA using the SNC patient software tool. Dimensions of isodose curve (ellipsoidal) was defined by major and minor diameters (i.e., *d*_1_ and *d*_2_) and corresponding diameter (*d*) of equivalent circle or sphere in a given volume was calculated as d = √ (*d*_1_ * *d*_2_). The equivalent diameters were compared between the TPS and measurements for further evaluation.

#### 2.1.4 Output factor constancy

Output factors were determined by the intermediate field method (IFM)^2^, ^3^ using the micro‐chamber (PTW 31021)^30^ and small field SRS diode (PTW 60018).^31^ The output factor constancy was investigated using the SRS diode (spatial resolution of 0.1 cm). The QA plans (with 200 MU) for the single field (at 0° gantry angle and 90° collimator angle) defining the fVC, including the fields with diminutive (± 0.5−1.0 mm) leaf shift, were generated for each energy beam and clinical setting. The corresponding dosimetric parameters (i.e., field output factors and dose/MU) were measured and dosimetric uncertainties caused by such infinitesimal leaf shift were examined. For instance, for each energy beam and clinical setting, dosimetric parameters of four different combination of fields (with a leaf shift ± 0.5  and ± 1.0 mm) were analyzed. The difference of dosimetric parameters between the TPS calculations and diode measurements were determined.^32^ Moreover, the dosimetric variation linked with potential fluctuations in MLC fields during the gantry rotation or arc treatment was quantified by considering a minute leaf shift and the dosimetric robustness of the fVC technique was also evaluated. Furthermore, the field output factors in small fields are specified as a function of the field size expressed by the equivalent square small‐field size (*S_clin_*), which is determined by explicitly measuring the FWHM of the profiles both in‐plane (X) and cross‐plane (Y) directions at the depth of dose measurements.^2^ For any rectangular small field, the equivalent square small‐field is given b:y^2^^,^ ^4^

(1) $$S_{clin\;}=\;\sqrt{FWHM\left(X\right)*FWHM\left(Y\right)}$$

### 2.2 Monte Carlo simulations

The EGSnrc Monte Carlo (MC) simulation package was used to simulate the Varian EDGE (Varian Medical Systems) linac.^33^ The MC simulations were performed in two steps: MC BEAM modelling of linac head with/without MLC using the BEAMnrc and 3D voxelized dose calculations in the phantom using the DOSXYZnrc. The MC beam model was designed including jaws (Y, X) and HD120 MLC by utilizing the phase‐space files (for 6 and 10 MV FFF beams) scored above the Y jaw those were provided by the vendor (Varian Medical Systems, Palo Alto, California, USA). Details of MC beam modeling provided in another article. The dose outputs (.3ddose files) from the DOSXYZnrc program were extracted and analyzed using the MATLAB 2020 (MathWorks.com) software. For the MC dose model validation, two‐dimensional (2D) dose profiles of 10 cm x 10 and 5 cm x 5 cm reference fields for both energy beams were compared between the MC simulations and Eclipse TPS.^34^, ^35^, ^36^ The comparison was made by recording the FWHMs of profiles scored in water phantom at 100 cm SSD. The corresponding depth dose curves were generated, and depths of maximum doses were recorded. Second, the dosimetric parameters such as TMR, OF were compared between the MC simulations and the TPS for static MLC fields of (10 × 10−0.5 × 0.5) cm^2^ at 95 cm SSD, 5 cm depth (100 cm SAD) in water phantom. Additionally, the OFs were compared with corresponding diode (PTW 60018) measurements for further validation. Ultimately, the potential dosimetric variation in very small (< 1 cm x 1 cm) MLC fields using fixed virtual cone method were analyzed amongst the MC, TPS and measurements (SRS diode) for the complete assessment. An extensive evaluation process of MC method will be described in a separate article.

## 3 RESULTS

### 3.1 Experimental

Results of all dosimetric measurements including the case study of 15 clinical TGN cases treated using the fVC method are presented in the following sections.

#### 3.1.1 Fixed virtual cone technique

The virtual cone beam model using the smallest (0.5 cm x 0.5 cm) but fixed HDMLC field is commissioned within 3% dose difference against measurements using the SRS MapCHECK and effectively implemented for the small‐target stereotactic radiosurgery of TGN patients (15 cases) at the clinic.

#### 3.1.2 Patient‐specific quality assurance

**FIGURE 4:**
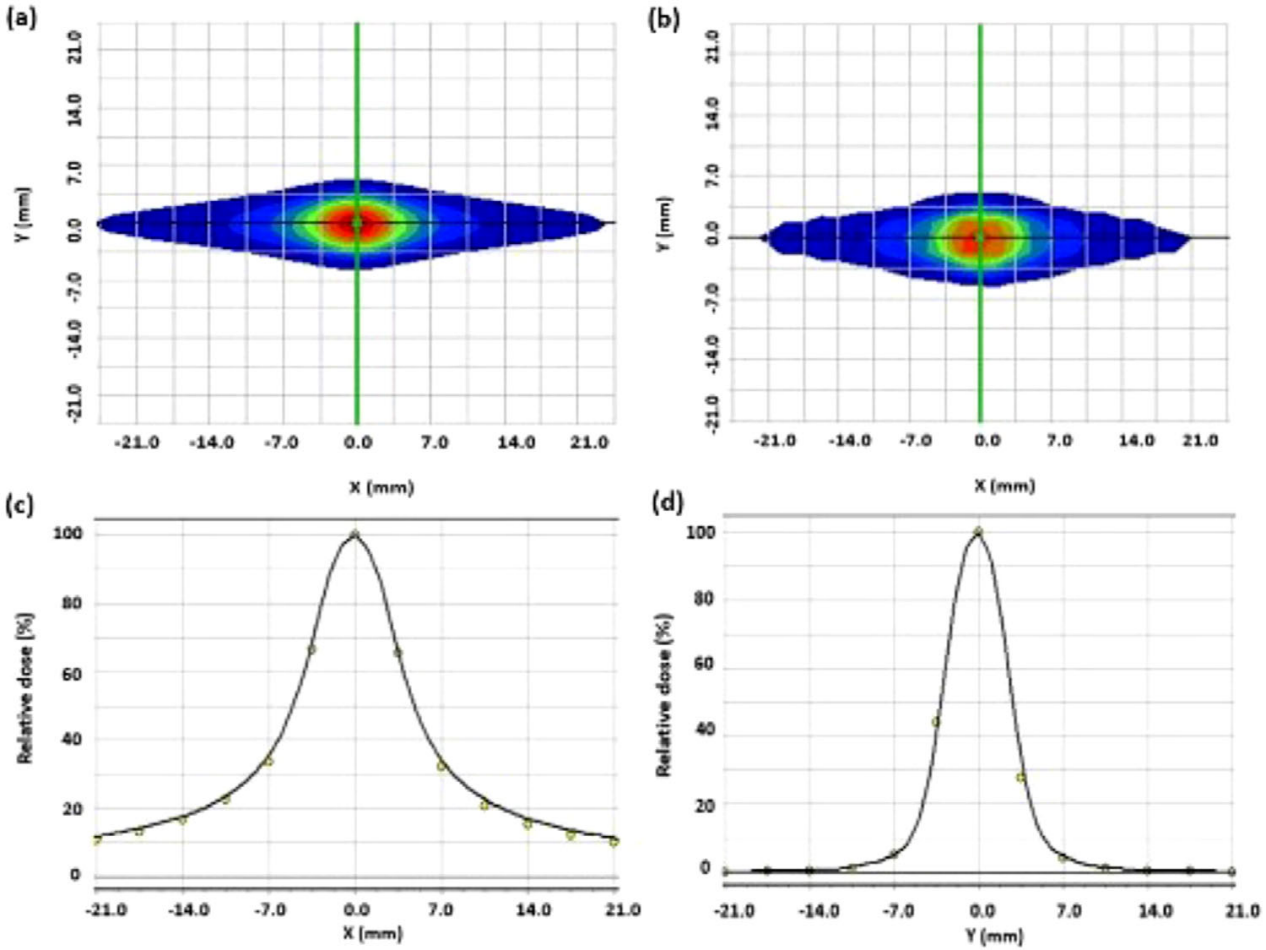
A representative case for gamma evaluation of treatment plans (10 MV FFF beam) for trigeminal neuralgia using the fixed virtual cone technique. Sections (a) and (b) represent planner dosemaps obtained from the TPS (Acuros XB) and SRS MapCHECK measurement, respectively. The sections (c) and (d) show the corresponding dose profiles in x and y directions, respectively. Solid line represents the planned dose profile, whereas yellow circle represents the measured one.

Table 1 shows results of the field‐by‐field study of 10 trigeminal cases (*n* = 130 fields) treated with the fixed virtual cone technique. The absolute value of average dose/MU difference between the SRS MapCHECK measurements and TPS for the 10 MV FFF beam was found (2.10 ± 1.30) % compared to (4.25 ± 1.80) % for the 6 MV FFF beam.^37^, ^38^ However, the difference in dose outputs (at central axis) was observed up to 12% for certain fields (arcs) depending on the gantry and collimator angles, energy beams, spatial resolution. The discrepancy was observed higher for the arc field with the gantry at 90°/270° and collimator at 0° angles. In addition, the comparison of whole treatment plans (i.e., cumulative dose comparison) resulted relatively higher passing rate (≥ 97%) for the 10 MV FFF beam compared to the 6 MV FFF beam (≥ 95%) with 2%/1 mm gamma criterion. The beam delivery time for 10 MV FFF plan was ∼ 5 min (∼ 30% decrease) shorter than for the 6 MV FFF plan. Furthermore, the measured dose profiles defined by S_clin_ are slightly wider than the planned ones and the apparent widening of field size are clearly visible (Figure 4c). For instance, the clinical field sizes (S_clin_) given by Equation (1) are reported as 0.57−0.60  and 0.60−0.64 cm for 0.5 cm fixed virtual cones of 6 and 10 MV FFF beams, respectively.

**TABLE 1:** Comparison of dose (cGy) per monitor unit (MU) between the TPS (Acuros XB) and measurements (SRS MapCHECK) during the field‐by‐field study of 10 treatment plans (average values of 130 fields) of trigeminal cases treated with the fixed virtual cone technique.

Dose (cGy)/ MU					
6 MV FFF			10 MV FFF		
Calculated	Measured	Difference (%)	Calculated	Measured	Difference (%)
0.45 ± 0.02	0.47 ± 0.02	4.25 ± 1.80	0.46 ± 0.02	0.47 ± 0.02	2.10 ± 1.30

#### 3.1.3 TGN cases study

Figure 4 represents the gamma evaluation for a trigeminal treatment plan (10 MV FFF beam) using the SRS MapCHECK, in which the cumulative absolute doses between the TPS and measurement are compared with 2%/1 mm gamma criterion. The isodose lines/curves are appeared ellipsoidal in shape due to the usage of various non‐coplanar arcs and variable MUs (i.e., it produces a constant dose per degree of the arc) during the treatment (Figure 4a and b). The average major diameters of the 90%, 50%, and 25% isodose curves are (2.1 ± 0.3) mm, (7.0 ± 0.8) mm, and (11.5 ± 1.2) mm, respectively. Whereas the respective minor diameters are (1.82 ± 0.25) mm, (5.7 ± 0.7) mm, and (7.5 ± 0.8) mm. The equivalent diameters (circle) of the 90%, 50%, and 25% isodose curves (coronal plane) for calculated and measured dose distributions are provided in Table 2.

**TABLE 2:** Comparison of calculated and measured equivalent diameters of 90%, 50%, and 25% isodose lines in coronal plane for 15 trigeminal cases treated with fixed virtual cone method.

Equivalent diameter^a^ (mm)					
90% isodose curve		50% isodose curve		25% isodose curve	
Calculated	Measured	Calculated	Measured	Calculated	Measured
1.95 ± 0.30	1.97 ± 0.20	6.10 ± 0.70	6.40 ± 0.50	8.70 ± 0.95	9.20 ± 0.80

^a^ The equivalent diameter is the diameter of a circle or a sphere of the given volume.

The calculated diameters are slightly smaller than the measured ones. The equivalent diameters of 50% isodose line are comparable to that generated using the 5 mm (diameter) physical cone. In Figure 4, the x‐dose profile (c) is wider than the y‐profile (d) due to a notable photon attenuation through MLC leaf edges and this effect is more dominant for the 10 MV FFF beam. The average clinical field size, given by Equation (1), for the SRS MapCHECK measurements was found as 6.4 mm using the fVC with 10 MV FFF beam. Additionally, the maximum dose delivered to the critical organ such as brainstem was reported ∼ 18 Gy (< 20 Gy) on average. The average volume of brainstem receiving maximum dose was 0.03 cm^3^.

#### 3.1.4 Output factor constancy

The output factor constancy was investigated with the SRS diode (PTW 60018) measurements using the intermediate field method and considering a static fVC field. Table 3 shows the comparison of dosimetric parameters (field output factors and dose/MU) between the calculations (TPS) and diode measurements for 0.5 cm x 0.5 cm fVC field of the 6 MV FFF and 10 MV FFF photon beams at (1) 95 cm SSD, 5 cm depth, and (2) 90 cm SSD, 10 cm depth.^37^, ^38^ The measured values are consistently higher compared to the calculated ones as expected. For 10 MV FFF beam, the absolute differences of field output factors between the calculated (TPS) and measured (SRS diode) were −1.26% and −1.00% at settings (1) and (2), respectively. Whereas such differences for the 6 MV FFF beam were −2.45% and −1.45% at respective settings. Similarly, difference in dose/MU for the 10 MV FFF beam were −1.27% and −1.20% at settings (1) and (2), respectively. Whereas the corresponding values were −3.35% and −2.56%, respectively, for the 6 MV FFF beam.

**TABLE 3:** Comparison of field output factors and Dose/MU between the Eclipse TPS (Acuros XB) and diode measurements (PTW 60018) for the 0.5 cm x 0.5 cm fixed virtual cone field (gantry at 0° and collimator at 90°) of the 6 MV FFF and 10 MV FFF photon beams at (1) 95 cm SSD, 5 cm depth, and (2) 90 cm SSD, 10 cm depth settings.

Beam + SSD/Depth	Field output factors			Dose (cGy)/MU		
	Calculated	Measured	Diff. (%)	Calculated	Measured	Diff. (%)
6 MV FFF 95/5 cm	0.605	0.620	2.45	0.558	0.577	3.35
10 MV FFF 95/5 cm	0.550	0.557	1.30	0.547	0.554	1.10
6 MV FFF 90/10 cm	0.560	0.568	1.42	0.424	0.435	2.56
10 MV FFF 90/10 cm	0.510	0.515	0.98	0.432	0.440	1.85

**FIGURE 5:**
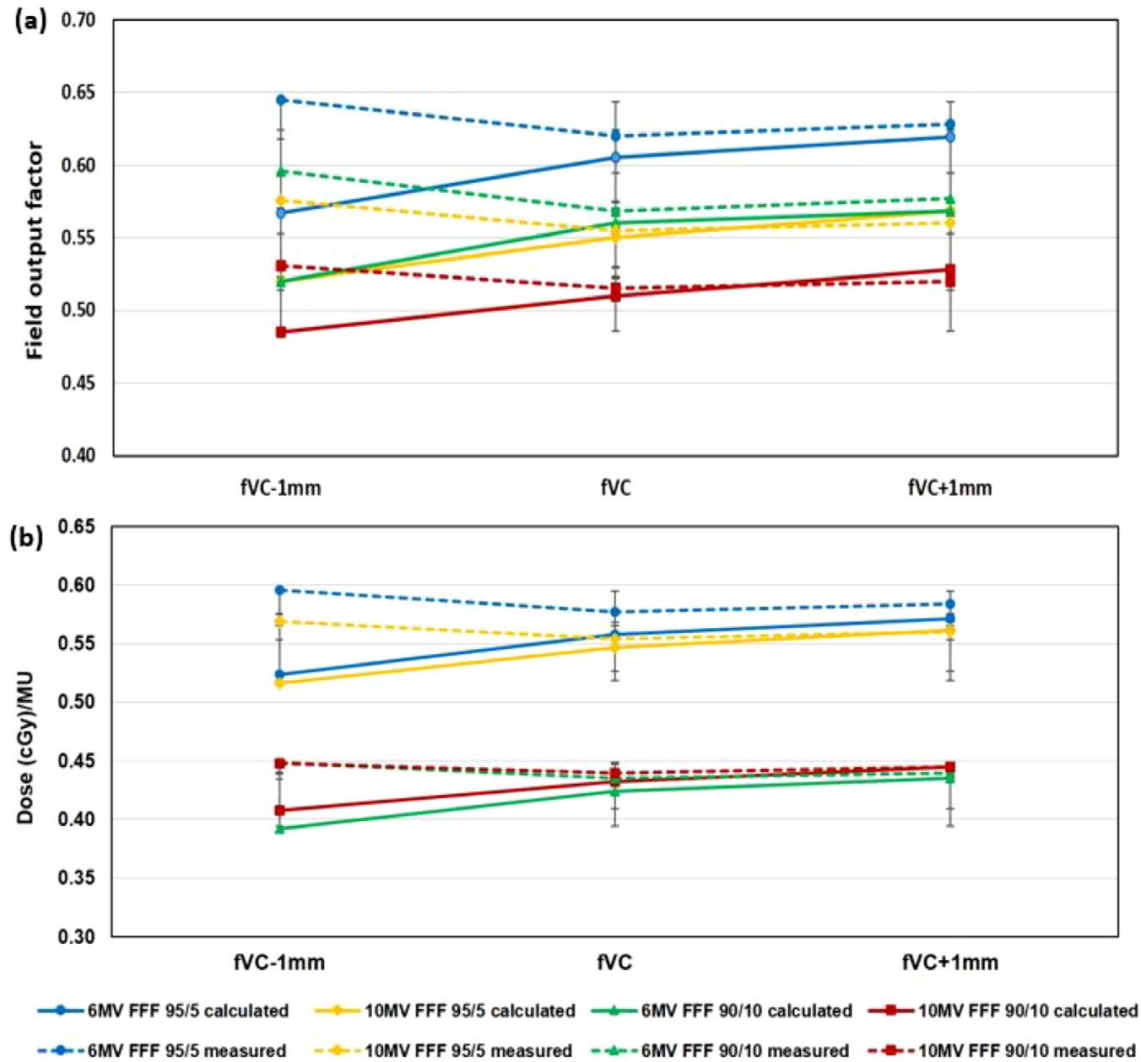
Comparison of (a) field output factors and (b) dose (cGy)/MU between the Eclipse TPS (Acuros XB) and SRS diode (PTW 60018) measurements for the field defining fVC (i.e., 0.5 cm x 0.5 cm HDMLC field defined with a gantry at 0° and collimator at 0°), including ± 1.0 mm leaf shift, for the 6 MV FFF and 10 MV FFF photon beams at different clinical settings. Solid and dotted lines represent calculated and measured parameters, respectively.

Furthermore, Figure 5 shows the comparison of (a) field output factors and (b) dose (cGy)/MU between the Eclipse TPS (Acuros XB) and SRS diode measurements (PTW 60018) for the field defining fVC, including ± 1.0 mm leaf shift, for the 6 MV FFF and 10 MV FFF photon beams at clinical settings (1) and (2). Solid lines represent calculated parameters in the fVC fields (including ± 1.0 mm leaf shift), whereas dotted lines display corresponding measured parameters. The calculated dosimetric parameters are found consistently lower (i.e., 5−10% for the 6 MV FFF beam and 3−5% for the 10 MV FFF beam, respectively) as compared to the corresponding measured parameters. In particular, the trend of these parameters seemed unusual (i.e., sudden fall off in calculated values whereas abrupt rise in measured ones) in fVC field with – 1.0 mm leaf shift exhibiting very high dosimetric uncertainties. Additionally, the calculated results exhibited notably higher (σ ≥ 4.0%) dosimetric variation (i.e., represented by error bars) in those non‐square or asymmetric MLC fields compared to measured ones (σ ≤ 2%). In other words, the margin of error or uncertainty (± 2σ) in the calculated field OF, dose per MU is significantly higher (∼ 4.5%) as compared to measured ones (∼ 2%). It is also observed that the clinical setting (2) has exhibited more accurate and consistent results than the setting (1) regardless of beam energy. Overall, the 10 MV FFF beam has delivered more stable dose outputs than the 6 MV FFF beam and the beam at setting (2) yielded better results.

### 3.2 Monte Carlo modeling

MC simulations were conducted for static fields with or without MLC and validated against the TPS. The optimal MC results (≤ 3% dose uncertainty and ≤ 3% dose difference to TPS) were obtained after simulating 30−50 millions of incident particles for 10 × 10 and 5 × 5 cm^2^ fields of both 6 MV FFF and 10 MV FFF beams at 100 cm SSD in water phantom.^39^ However, the dose uncertainty was slightly increased for the smaller fields but did not exceed 5% threshold. Detailed results of the model validation will be presented in a separate research paper.

The MC study of fVC included simulations of the 0.50 cm x 0.50 cm HDMLC field and evaluation of dosimetric variation due to a minute leaf shift (± 0.5−1.0 mm) in the field. The equivalent square field size (*S_clin_*), given by Equation (1), of the MC simulated dose profiles were recorded slightly higher than that obtained from the TPS. Particularly, for nominal square field sizes of 0.50 cm, *S_cli_*_n_ were 0.60 cm for MC as compared to 0.57 cm from TPS using 6 MV FFF beam, whereas 0.62 cm for MC versus 0.59 cm of TPS using 10 MV FFF beam. Additionally, the comparison field output factors has shown a reasonable (≤ 4% difference) agreement against measurements thereby the 10 MV FFF beam yielding comparatively better results than the 6 MV FFF beam. Furthermore, results of MC study for dosimetric (uncertainty) evaluation in the fVC fields including ± 1.0 mm leaf shift have shown similar (pattern) dosimetric variation (σ ≤ 2.5%) as the measured ones (σ ≤ 2.0%).^38^ However, the model requires further investigation for comprehensive results.

## 4 DISCUSSION

### 4.1 Experimental

During the PSQA, the dose (at central axis) difference was observed notably higher (up to 12%) for certain fields (arcs) depending on the gantry and collimator angles, energy beams, which is on par with previous studies.^18^, ^19^, ^22^, ^40^ Note that the highest dose deviation was associated with the arc field when the gantry at 90°/270° and collimator at 0° angles that can be avoided or corrected during treatment planning.^9^, ^19^, ^40^, ^41^ Our approach minimizes these inconsistencies by utilizing a symmetric MLC field with a fixed jaws setting and using various non‐coplanar arcs with minimal error so that it is capable of delivering more accurate and precise dose to the target. Regarding dosimetric uncertainty analysis in fVC field, the 6 MV FFF beam exhibited relatively higher difference in outputs between measurements and TPS (Table 3) along with a larger variation (Figure 5) than 10 MV FFF beam, which in on par with previous studies.^3^, ^27^, ^31^, ^42^ Since dosimetric uncertainty increases (up to 5%) with decrease in field size upto 0.5 cm x 0.5 cm and discrepancy between calculated and measured parameters rises notably due to the small field, Acuros XB commissioning, and diode measurements related uncertainties.^2^, ^42^, ^43^ When the field size < 1 cm x 1 cm, Dosimetric uncertainties tend to increase rapidly and become more challenging to quantify.^2^, ^4^ It is obvious that the higher deviation (Figure 5) in calculated values in the fVC fields (± 1.0 mm shift) and larger difference compared to measurements are fairly reasonable results. In addition, comparatively lower values for the 10 MV FFF beam than the 6 MV FFF beam indicate that more particles escape the field due to a larger lateral range of the secondary charged particles and the higher transmission through the penumbra for higher energy beams.^2^, ^4^ An overall dosimetric inconsistency in the fields with ± 0.5 mm leaf shift are found to be insignificant (difference ≤ 3%). Whereas it is noteworthy (up to 12% difference) with ± 1.0 mm shift. These results are consistent with the field‐by‐field study during the quality assurance process specifically for arc fields with the gantry at 90° or 270° and the collimator at 0° angles. Early estimation of dosimetric impact due to a tiny fluctuation in MLC field/leaf during the gantry or collimator rotation could give a flexibility of applying corrections in treatment planning if necessary.

Additionally, our method allows freedom to manipulate the dose delivery (i.e., with selective arcs, arc span, and arc weight, variable MUs) such that the tumor receives maximum dose and critical organs receive minimal dose. As the dose per degree of the arcs is usually non‐uniform and proportional to the sine of the gantry angle.^15^, ^22^ The weighting is allocated in such a way that it results uniform fluence over an area that is ideal for generating a spherical dose distribution. Due to the constancy of dose per degree of arcs, the isodose lines/curves for trigeminal treatment plans are appeared to be ellipsoidal with significant dose spill in anterior‐posterior direction as illustrated in Figure 4a, b. A critical advantage of generating such dose distributions is to maximize the dose to the target while minimizing that to the critical organ (brainstem).^15^, ^16^, ^22^ Furthermore, the 10 MV FFF beam was chosen because it can deliver higher dose rate (up to 2400 MU/min) thereby minimizing the treatment time (∼ 5 min) and it also provides more dosimetric stability and reliable dose delivery.^15^ Automated table motion during the beam delivery will further reduce treatment time thereby making our technique even more efficient. The dimensions (i.e., major and minor diameters) of 90%, 50%, and 25% isodose lines (ellipsoidal) are manually obtained from the treatment plans and measurements. The equivalent diameters of the corresponding circles or spheres of given volume are computed to approximate the prescribed, irradiated volumes, dose coverage, proximity of critical organ (brainstem) as reported in Table 2. The dose distribution (50% isodose line) of fVC is equivalent to 5 mm physical cone. Additionally, average treatment time is ∼ 45 min, including initial set up, cone beam CT (initial, mid treatment), beam delivery (∼ 20 min), which is comparable to previous studies.^28^, ^29^, ^30^ The maximum dose delivered to the brainstem was restricted to < 25 Gy (average ∼ 18 Gy).

### 4.2 MC modeling

Although we discuss details of the MC results in a separate paper. It is worth mentioning that the MC calculated parameters (i.e., *S_cli_*_n_, field output factors) for the 0.5 cm x 0.50 cm fVC field are found relatively larger compared to the TPS, but smaller than measured for smaller fields, which is on a par with previous studies.^2^, ^3^ For instance, MC simulated *S_clin_* was 0.60 cm for 10 MV FFF beam and 0.58 cm for 6 MV FFF beam, respectively. Whereas respective field OFs were 0.562 and 0.511, calculated at 90 SSD, 10 cm depth. It is due to the fact that the apparent widening effect becomes pronounced in very small fields^5^ and even more for higher energy beams.^4^ It means more particles can escape the field due to a larger lateral range of the secondary charged particles and the higher transmission through the penumbra for higher energy beams. The potential sources of the larger dosimetric uncertainties as well as discrepancies reported in very small fields could be due t:o 2 ^,^ ^4^, ^34^, ^35^, ^44^ (1) the presence of prominent small‐field conditions, (2) an inaccurate modeling of geometry (MLC), (3) the adoption of different dose calculations modalities (i.e., a volume‐based dose calculation in MC versus a point‐based dose calculation in TPS), and (4) the difference in source type (i.e., energy spectrum or fine tuning versus monoenergetic beam).

Furthermore, we extended the MC study to evaluate dosimetric uncertainties in fixed virtual cones with ± (0.5−1.0) mm leaf shift. Early MC results (water phantom) demonstrate the dosimetric variation (pattern) are more aligned to measurements (SRS diode) rather than TPS ones, which indicates the MC fairly estimates (σ ∼ 2%) the uncertainties in these asymmetric fields, even better than the TPS (σ ∼ 4%) when TPS only uses symmetric fields.^37^, ^38^ It could result a significant dosimetic inconsistencies during small‐field SRS arc treatments using dynamic MLC fields unless these are properly addressed in advance. These results justify the importance of MC study specifically for smaller fields besides the TPS and measurements. However, the MC model needs further improvements that can be done with rigorous simulations by inserting the accurate geometrical (i.e., MLC, detector, and phantom) details for the complete assessment. Lately, the MC dose uncertainty has been further reduced (≤ 3%) after applying various variance reduction techniques and extensive simulations^44^, ^45^ and results are being evaluated quantitatively, which will be discussed elsewhere.

Overall, the 10 MV FFF beam has shown better performance than the 6 MV FFF beam in both clinical settings and our test setting of 90 cm SSD, 10 cm depth with more favorable results. These results substantiate that our fixed virtual cone with 10 MV FFF beam is a straightforward and reliable method, which can be effectively implemented in routine small target radiosurgery of TGN.

## 5 CONCLUSION

The fixed virtual cone technique using the 0.5 cm x 0.5 cm HDMLC field for small‐targeted stereotactic radiosurgery such as TGN is successfully validated within 3% dose difference against measurements using the SRS MapCHECK. As the quality assurance measure, the comparison of dosimetric parameters (field output factors, dose/MU) between the TPS and SRS MapCHECK measurements has shown relatively better agreement for the 10 MV FFF beam compared to the 6 MV FFF beam. The output factors constancy test using the TPS and SRS diode measurements for the fVC fields including a minute leaf shift (± 0.5−1.0 mm) has shown a notable (3−10%) difference along with 1.5−4.5% uncertainty, depending on beam energy and clinical settings. Furthermore, early results of independent MC study for the fVC fields have shown similar (trend) dosimetric variation as measured but relatively better than TPS results. However, the model can be improved further by simulating the accurate geometrical (i.e., MLC, detector, and phantom) details for the final assessment. Collectively, the fVC with 10 MV FFF beam has yielded more accurate and consistent results.

The feasibility of the fixed virtual cone technique for a small‐targeted, high‐precision stereotactic radiosurgery and its dosimetric robustness due to a potential leaf drift during the gantry rotation are evaluated using measurements and MC simulations. This is a convenient and reliable alternative to the physical cone (5 mm), which can be routinely applied for the SRS of trigeminal neuralgia. This research can be extended further by a machine learning approach based on MLC log files as well as radiomics analysis from QA images.

## AUTHOR CONTRIBUTIONS

Taindra Neupane has substantial contributions to the conception or design of the work, or acquisition, analysis, interpretation of data for the work. He has drafted/revised it critically and makes a final decision for the approval of final version. The author agrees hold the accountability for all aspects of work in ensuring that questions related to the accuracy or integrity of the work.

Charles Shang has major contributions to the conception or design of the work and acquisition/analysis/interpretation of data for the work. He has revised it critically and makes a final decision for the approval of final version. The author agrees hold the accountability for all aspects of work in ensuring that questions related to the accuracy or integrity of the work.

Maxwell Kassel has considerable contributions to the acquisition/interpretation of data for the work. He has revised the draft critically as a native speaker and suggests in the decision making for the approval of final version. The author agrees hold the accountability for all aspects of work in ensuring that questions related to the accuracy or integrity of the work.

Wazir Muhammad has useful contributions to the interpretation of data for the work. He has contributed notably towards the scientific revision of the draft and supports in the decision making for the approval of final version. The author agrees hold the accountability for all aspects of work in ensuring that questions related to the accuracy or integrity of the work.

Theodora Leventouri has valuable contributions to the design of the work. She has contributed significantly towards the scientific revision of the draft as a native speaker and supports in the decision making for the approval of final version. The author agrees hold the accountability for all aspects of work in ensuring that questions related to the accuracy or integrity of the work.

Timothy R. Williams has valuable contributions to the inception, concept and design of this treatment technique and has a critical role in its implementation into the clinic. He has contributed notably towards the sceintific revision of the draft and gives the final decision for approval. The author agrees to hold accountability for all aspects of work in ensuring that questions related to the validity and integrity of the work.

## CONFLICT OF INTEREST STATEMENT

The authors declare no conflicts of interest.
